# Peptide-Rich Yeast Fractions from Brewer’s Spent Yeast: A Scalable Fractionation Approach and Their Functional Application in Bakery Products

**DOI:** 10.3390/foods14071144

**Published:** 2025-03-25

**Authors:** María Emilia Brassesco, Ana Paupério, Carlos D. Pereira, João Paulo Ferreira, Manuela Pintado

**Affiliations:** 1 Universidade Católica Portuguesa, CBQF—Centro de Biotecnologia e Química Fina—Laboratório Associado, Escola Superior de Biotecnologia, Rua Diogo Botelho 1327, 4169-005 Porto, Portugal; airibeiro@ucp.pt (A.P.); jpferreira@ucp.pt (J.P.F.); mpintado@ucp.pt (M.P.); 2 Politécnico de Coimbra, Escola Superior Agrária, Bencanta, 3045-601 Coimbra, Portugal; cpereira@esac.pt

**Keywords:** bioactive compounds, ultrafiltration process, process scale-up, functional ingredients, by-product valorization

## Abstract

Brewer’s spent yeast (BSY), a significant brewing by-product, poses environmental challenges and opportunities for valorization as a sustainable protein source. This study focuses on transforming BSY into high-value functional ingredients for food applications. A green, sustainable, and scalable process was developed to extract bioactive compounds from BSY at both laboratory and pilot scales, yielding peptide-rich fractions with robust antioxidant properties. These extracts were incorporated into prototype formulations, including protein-enriched crackers, demonstrating their potential as natural, nutritious ingredients. Physicochemical, compositional, and functional characterizations validated their application viability. The antioxidant potential of BSY fractions was confirmed through total phenolic compounds and ABTS and oxygen radical absorbance capacity assays, where the retentate from the 10 kDa ultrafiltration fraction on the pilot scale exhibited superior bioactivity, supporting its selection as the most suitable fraction for food formulations. Additionally, the transition from laboratory to pilot scale revealed slight variations in protein retention and bioactive compound recovery, emphasizing the need for process optimization. These findings highlight BSY’s potential to support circular economy practices by reducing waste while enhancing the nutritional and functional value of food products.

## 1. Introduction

The increasing global demand for sustainable food systems has intensified the need to valorize agro-industrial by-products, promoting both environmental and economic benefits. Among these by-products, brewer’s spent yeast (BSY) has emerged as a promising resource due to its high nutritional value, containing proteins, β-glucans, nucleotides, vitamins, and minerals [1,2]. BSY is collected through sedimentation during the final stage of the second fermentation and beer maturation, just before full maturation. The excess yeast can then be harvested and reused for up to six cycles, maximizing its utility in brewing [3]. With the BSY market projected to reach $2.17 billion by 2030, this by-product presents a valuable opportunity for both waste reduction and protein replacement [4]. Currently, BSY is utilized in various industries, including animal feed as a protein supplement, biofuel production, and as a raw material in cosmetics, highlighting its diverse applications and economic potential [5,6,7]. However, more research is needed to optimize its use in human food products.

The direct utilization of BSY in human food systems has been limited due to both sensory and functional challenges. BSY contains high levels of ribonucleic acid (RNA), and excessive intake can lead to elevated uric acid levels in the bloodstream, increasing the risk of gout [5]. Moreover, the presence of bitter compounds and off-flavors significantly hinders its acceptance in food products. To overcome these challenges, thermal and enzymatic treatments have been applied to degrade and remove RNA, improving both the sensory and functional properties of BSY [5]. Additionally, innovative biotechnological approaches have facilitated the fractionation and extraction of bioactive peptide-rich extracts from BSY, alongside β-glucans and mannan-oligosaccharides, broadening its potential applications across multiple industries [8,9,10]. Studies have demonstrated that BSY-derived peptides display a range of functional properties, including strong antioxidant activity, due to their capacity to scavenge free radicals and inhibit lipid peroxidation [9], as well as their significant antihypertensive potential through angiotensin-converting enzyme (ACE) inhibition [11]. Moreover, specific peptides isolated from BSY hydrolysates have shown antiulcer and antiproliferative activities, reinforcing their role as functional bioactive components suitable for incorporation into health-promoting foods [12]. The composition of these peptides is enriched in amino acids, such as leucine, valine, and proline, which contribute to their structural stability and interaction with biological targets [10]. In parallel, β-glucans extracted from BSY have been successfully incorporated into functional foods like cereals, yogurts, and beverages, enhancing their nutritional profiles and market appeal [13,14]. These findings highlight the potential of BSY peptide fractions to be used in nutraceuticals, protein-enriched formulations, and functional food ingredients, making them valuable components in the development of health-oriented food products [15].

The development of protein-enriched crackers has gained significant interest as a strategy to enhance the nutritional profile of conventional bakery products. Traditional wheat-based crackers are primarily rich in carbohydrates but low in protein and fiber, making them a suboptimal choice for consumers seeking functional foods. Recent research has explored the incorporation of alternative protein sources into cracker formulations, such as insect flour [16] and plant-based proteins like faba bean isolates [17]. These studies highlight the potential of non-conventional protein sources in enhancing nutritional quality, improving amino acid balance, and even influencing textural and sensory properties.

The work by Amorim et al. [18] demonstrated that selective membrane filtration, combined with enzymatic hydrolysis, effectively preserves and enhances the functional potential of these fractions, making them suitable for applications in the food and nutraceutical industries. However, considering the findings of Oliveira et al. [10], who compared various extraction methodologies—including high-pressure homogenization, sonication, autolysis, and enzymatic hydrolysis—our study opted for autolysis as a more sustainable and efficient approach. Oliveira et al. [10] concluded that autolysis offers significant advantages in terms of biomass and water consumption while still yielding high protein content and a substantial proportion of low-molecular-weight peptides. This aligns with the growing need for green and scalable processing techniques, reducing the reliance on external enzymatic inputs. By employing autolysis as the sole extraction method, our study ensures a more environmentally friendly and cost-effective approach to obtaining BSY-derived biopeptides while maintaining their functional integrity.

The valorization of agro-industrial by-products as functional ingredients in food formulations has gained considerable attention due to their high nutritional value and contribution to circular economy principles [19,20]. By-product flours derived from fruits and cereals, such as wheat germ and tomato pomace, are particularly rich in dietary fiber, protein, and bioactive compounds, making them excellent candidates for improving the nutritional profile of bakery products [19,21]. Wheat germ, a milling co-product, is an abundant and underutilized ingredient containing up to 30% protein, essential fatty acids, and dietary fiber, along with significant amounts of vitamin D and antioxidant compounds, such as tocopherols and polyphenols [22]. Given its high-quality protein composition, wheat germ serves as a sustainable protein alternative while also contributing to the fiber content in food formulations [22]. Tomato pomace, a fiber-rich by-product of tomato processing, has been widely recognized for its high levels of soluble fiber, polyphenols, carotenoids (lycopene), and prebiotic potential, which enhance both the nutritional and functional properties of food products [19,23,24]. Studies have shown that fiber fractions from tomato residues can improve gut health, modulate microbiota composition, and increase antioxidant capacity when incorporated into food matrices [25]. Furthermore, the presence of pectin and hemicelluloses in tomato pomace contributes to improved texture and water-holding capacity in baked goods, enhancing their overall sensory properties [26]. Cream crackers enriched with tomato pomace have already been studied and have shown better physical and nutritional characteristics [27].

So, this study explores a scalable process for obtaining BSY-derived fractions through ultrafiltration and evaluates their functional potential in food applications. Six BSY fractions were obtained at laboratory and pilot scales, targeting bioactive peptides and functional compounds. Crackers were chosen as a model food to assess the feasibility of BSY incorporation, aiming to enhance protein content and antioxidant properties. Additionally, tomato pomace was included to improve the physical and nutritional (fiber and essential nutrients) properties, and wheat germ was also included to contribute protein, creating a nutritionally balanced formulation [28]. The study aims to (i) refine the BSY fractionation process, (ii) compare laboratory and pilot-scale yields and bioactivity, and (iii) evaluate the functional and nutritional properties of BSY-enriched crackers. By establishing a direct link between BSY fractionation and food formulation, this work provides insights into utilizing protein-rich yeast fractions as innovative ingredients in functional bakery products supporting circular economy practices by reducing waste.

## 2. Materials and Methods

### 2.1. Reagents

ABTS diammonium salt (2,2-azinobis-3-ethylbenzothiazoline-6-sulfonic acid), 2,20-azobis (2-methylpropionamidine) dihydrochloride (AAPH), fluorescein, methanol, potassium sorbate, and sodium carbonate were purchased from Sigma-Aldrich (Sintra, Portugal). Gallic acid monohydrate was purchased from Sigma-Aldrich (St. Louis, MA, USA). The Folin-Ciocalteu reagent and D-galactose (CAS 59-23-4) were purchased from Merck KGaA (Darmstadt, Germany). Methanol ≥ 99.9% was purchased from Fisher Scientific (Loughborough, UK). The ultrapure water was obtained from the Milli-Q^®^ Advantage system, Merck KGaA (Darmstadt, Germany).

### 2.2. Tomato Pomace By-Product and Wheat Germ

The tomato pomace used in this study was kindly provided by the Competence Center for the Tomato Industry (CCTI) and subsequently processed at the Agri-Food Technology Support Center (CAATA). A total of 100 kg of tomato pomace was collected and processed, following previously established conditions at the Universidade Católica Portuguesa (UCP). The transformation process involved centrifugation to remove excess moisture, followed by a drying step (by convection) to achieve a final moisture content of 6% for stabilization and powdered ingredient production. However, due to the naturally low moisture content in the collected tomato pomace, only the drying step was required. Finally, the dried material was ground and fractionated using an automatic sieve shaker (Retsch AS 200, Haan, Germany) with circular oscillation, with 50 g of flour sifted for 5 min at an amplitude of 1.85 into three particle size ranges: (1) <100 µm, (2) <250 µm, and (3) >250 µm, with sieves of 20 cm in diameter. In this study, the fraction with a particle size of <250 µm was selected, as this range is recommended for food applications due to its improved textural properties and digestibility. Larger particle sizes typically contain higher amounts of insoluble fiber, which can negatively impact sensory attributes and limit bioavailability [29].

The wheat germ was kindly provided by the company Germen Moagem de Cereais. It was dried overnight at 60 °C in a UN450-PLUS Natural Convection Lab Oven (Memmert^®^, Büchenbach, Germany, 449L) and stored in vacuum-sealed bags until use. The chemical characterization of both by-products was conducted, with detailed methodologies outlined in Section 2.5.

### 2.3. BSY By-Product

The BSY used in this study was kindly provided by the Super Bock Group (Porto, Portugal). The chemical characterization of the initial BSY by-product was conducted, with detailed methodologies outlined in Section 2.5. The processing was performed at both laboratory and pilot scales, starting with autolysis at 70 °C for 5 h in 1 L and 100 L vessels, respectively. At the laboratory scale, before undergoing autolysis, the BSY was first subjected to two consecutive washing and centrifugation steps (8000 rpm, 10 min, and 4 °C) to remove residual beer. Following this pre-treatment, 800 mL of BSY was mixed with water at a 1:2 ratio (BSY:H_2_O) and autolyzed using a Smart Cooking Robot (Xiaomi, Beijing, China) at level 1 [10]. At the pilot scale, autolysis was performed in a double-walled, steam-supplied vat with controlled stirring and temperature regulation. Unlike the laboratory scale, no additional water was added at this stage, and the yeast underwent direct autolysis using its inherent moisture content [18]. The resulting supernatants from both scales were immediately processed through a membrane filtration system (see the following section).

### 2.4. Optimization and Scale-Up of BSY Fractionation Processes

**Figure 1:**
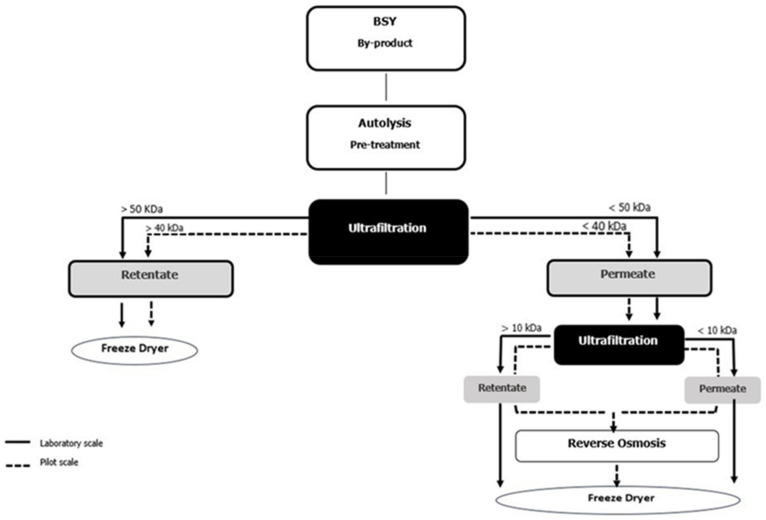
Diagram of the scale-up membrane filtration processes.

The BSY autolysates from laboratory and pilot-scale processes were subjected to sequential selective membrane filtration to obtain distinct protein and peptide fractions (BSY extract). The workflow of the filtration processes is outlined in Figure 1. At the laboratory scale, 700 mL of BSY:H_2_O autolysate underwent tangential flow filtration using the Cogent^®^ µScale TFF system, with membranes having molecular weight cut-offs (MWCO) of 50 kDa and 10 kDa (Millipore, Jaffrey, NH, USA). The filtration was conducted at 25 °C, with a transmembrane pressure of 4 bar and a flow rate of 0.1 L/min. Prior to filtration, the autolysate was centrifuged at 8000 rpm for 10 min at 4 °C to remove insoluble material. Following filtration, the permeate and retentate fractions were collected separately and freeze-dried in a basic research freeze dryer (LyoAlfa, Telstar, Barcelona, Spain).

In contrast, in the pilot-scale process, 100 L of autolysate were fractionated using an organic ultrafiltration (UF) membrane (UF SD 3838/30 FDA 40 kDa, SIVE Fluid Systems, Madrid, Spain) with a 7.0 m^2^ filtration area and a 40 kDa cut-off, operated at 45–50 °C and a transmembrane pressure of 3.5–4.0 bar. The UF permeate (UFP40, MW < 40 kDa) underwent further ultrafiltration using a 10 kDa organic membrane (UF SD 3838/30 FDA 10 kDa, SIVE Fluid Systems, Madrid, Spain) with a 7.0 m^2^ filtration area under the same temperature and pressure conditions. The ultrafiltration retentate and ultrafiltration permeate (P/RUF10, MW > 10 kDa and P/PUF10, MW < 10 kDa) were concentrated by reverse osmosis using a pilot plant unit ORM (Amadora, Portugal) equipped with a 2.5 S Seawater pressure vessel and a 1 m^2^ Filmtec membrane SW302540 (Dow Chemical Company, Midland, TX, USA), and then freeze-dried under the same conditions as P/RUF40 and P/PUF10 in a Lyph-Lock freeze dryer (Labconco Corporation, Kansas City, MI, USA).

Finally, a total of six distinct fractions were collected from the two scales:

- Laboratory-Scale Fractions:
  ○ L/RUF50: retentate from 50 kDa ultrafiltration.
  ○ L/RUF10: permeate from 50 kDa/retentate from 10 kDa ultrafiltration.
  ○ L/PUF10: permeate from 10 kDa ultrafiltration.
- Pilot-Scale Fractions:
  ○ P/RUF40: retentate from 40 kDa ultrafiltration.
  ○ P/RUF10: permeate from 40 kDa/retentate from 10 kDa ultrafiltration.
  ○ P/PUF10: permeate from 10 kDa ultrafiltration.

All fractions were subsequently freeze-dried and stored under vacuum at ambient temperature, protected from light to preserve their functional properties. The yield (% DW) of each fraction obtained was calculated using the following formula:

(1) $$Yield\;\left(\%\right)=\frac{\mathrm{Weight}\;\mathrm{of}\;\mathrm{the}\;\mathrm{dried}\;\mathrm{fraction}\;\mathrm{obtained}}{\mathrm{Initial}\;\mathrm{dry}\;\mathrm{weight}\;\mathrm{of}\;\mathrm{BSY}\;\mathrm{used}}\times 100$$

For the laboratory scale, the initial dry weight processed was 36.177 g DW, and for the pilot scale, the initial dry weight processed was 29.956 kg DW. This allowed for an accurate assessment of the recovery efficiency for each BSY fraction throughout the process.

### 2.5. Nutritional Composition

All procedures followed the guidelines outlined in the Official Methods of Analysis [30], the International Organization for Standardization [31,32], or the relevant Portuguese regulation [33]. The crude protein content was determined using the Kjeldahl method [32]. A conversion factor of 5.6 was applied to account for the high non-protein nitrogen content in yeast extracts [10,34], while a factor of 6.25 was used for crackers. The lipid content was determined following the AOAC method 920.39. The crude ash content was estimated through incineration [33]. The moisture content was determined according to the [31] method. The total dietary fiber (TDF) content was calculated using the enzyme–gravimetric method, according to the AOAC method 991.43 (1990), with slight modifications [35]. The energy contents were computed using the general Atwater factors (4 kcal/g for protein, 4 kcal/g for carbohydrates (without the total fiber), 9 kcal/g for fat, and 2 kcal/g for total fiber contents), and the results were expressed in kcal per 100 g of the corresponding sample on a dry basis (kcal/100 g DW) [36]. An optical emission spectrometer Model Optima 7000 DV ™ ICP-OES (Dual View, PerkinElmer Life, and Analytical Sciences, Shelton, CT, USA) with radial configuration was used for minerals analysis. A calibration curve of commercial mix standards for ICP analysis (Inorganic Ventures, Christiansburg, Christiansburg, VA, USA) (molybdenum, zinc, cadmium, phosphorus, lead, nickel, cobalt, boron, manganese, iron, magnesium, calcium, copper, aluminum, sodium, and potassium) from 0.05 to 10 mg/L was applied for quantification. Microwave digestion was performed before ICP analysis in a Speedwave XPERT (Berghof Products + Instruments GmbH, Eningen, Germany) using 500 mg of the sample with 10 mL of Suprapur^®^ HNO_3_. The percentage of daily recommended intake (%DRI) for each mineral based on EFSA’s Population Reference Intake (PRI) values was calculated using the following formula:

(2) $$DRI\;\left(\%\right)=\frac{\mathrm{Mineral}\;\mathrm{content}\;\mathrm{per}\;100g\;\mathrm{DW}}{\mathrm{PRI}}\times 100$$

All measurements were conducted in triplicate and expressed as grams per 100 g on a dry weight (DW) basis.

### 2.6. Water- and Oil-Holding Capacity (WHC and OHC)

The water- and oil-holding capacities of each laboratory and pilot fraction were determined by centrifugation, following the method described in [37]. The oil-holding capacity (OHC) was assessed under the same conditions as WHC, using vegetal oil and expressed as grams of oil retained per gram of sample.

### 2.7. Color Measurement

The color was analyzed from the six distinct fractions. A portable CR-410 Chroma meter (from Minolta Chroma, Osaka, Japan) was used to determine the color point. The CIELAB color system (L*, a*, and b*) was used to determine the color point, in which the L* corresponds to the luminosity coordinate (an L* value of 100 for a white object and 0 for a black object), the a* sets the green–red coordinate, and the b* sets the blue–yellow color coordinate [38].

### 2.8. Water Activity (a_w_) Measurement

The water activity of the cracker samples was determined in a LabMaster-a_w_ Neo (Novasina AG, CH-8853, Lachen, Switzerland). The reading temperature was around 25 °C. Three replicates were performed for each experiment.

### 2.9. Peptide Profile

#### Molecular Weight Distribution

High-Performance Size Exclusion Chromatography (HPSEC) was conducted following the methodological approach detailed by Fernandez Cunha et al. (2023) [39]. Each sample was injected at a volume of 10 μL, and prior to analysis, all samples were filtered through PTFE/L 0.22 μm membranes to ensure purity and prevent particulate interference.

### 2.10. Bioactivity Characterization

Samples were analyzed based on their polyphenol content and corresponding antioxidant activity.

#### 2.10.1. Extraction of Phenolic Compounds

Phenolic compounds from the BSY fractions and cracker prototypes were extracted with 80% ethanol at a 1:20 (*w*/*v*) ratio, following the protocol outlined by León-González et al. [40], with some modifications. In the case of the crackers, they were milled into a fine powder using a BOSCH TSM6A013B coffee grinder (Stuttgart, Germany) before proceeding with the extraction protocol. The mixtures were incubated overnight at room temperature on an orbital shaker (200 rpm) to enhance extraction. Subsequently, the samples were centrifuged at 8000 rpm for 20 min, and the resulting supernatant was collected for the analysis of total phenolic content and antioxidant activity.

#### 2.10.2. Total Phenolic Content

##### Folin–Ciocalteu Assay

The total phenolic content (TPC) was determined by the Folin–Ciocalteu colorimetric method described in [41], with some modifications. In a 96-well microplate Reader Biotek Synergy H1-1 (Agilent Technologies, Inc., Santa Clara, USA), 30 µL of each digested sample (diluted in methanol) was mixed with 100 µL of Folin–Ciocalteu reagent (20% *v*/*v*) and 100 µL of anhydrous sodium carbonate solution (7.4% *w*/*v*). The control used was methanol. A standard curve was determined using different concentrations of gallic acid (0.025–0.2 mg/mL). The microplate was incubated at 25 °C for 30 min, and the absorbance of the resulting blue mixtures was measured at 765 nm. The TPC values were expressed in mg of gallic acid equivalents (GAEs) per mL of sample.

#### 2.10.3. Antioxidant Activity

Antioxidant activity (AA) was measured using the ABTS (2,2-azinobis-(3-ethylbenzothiazoline-6-sulphonic acid)) and the oxygen radical absorbance capacity (ORAC) assays. All analyses were performed in triplicate and expressed in mg of Trolox equivalents (TEs)/g DW. All these assays were performed using the Synergy H1 multidetection plate reader (BioTek Instruments, Winooski, VT, USA), operated with Gen5 BioTek software version 3.04.

##### ABTS Assay

The ABTS method, as described by Gonçalves et al. [42], determined the total antioxidant activity of all the fractions and crackers with some modifications. ABTS was dissolved in water at a 7 mM final concentration. The ABTS radical cation ABTS•+ was produced by reacting ABTS stock solution with potassium persulfate (Merck) (final concentration of 2.44 mM) and kept in the dark at room temperature (25 ± 2 °C) for 12–16 h before use. Before the analysis, (ABTS•+) was filtered using a 0.22 μm filter (Orange Scientific, Braine-l’Alleud, Belgium) and diluted in methanol to an absorbance of 0.700 ± 0.02 at 734 nm. In a 96-well microplate, 20 µL of the digested sample was mixed with 180 µL of the ABTS•+ working solution. The control used was methanol. A standard curve was determined using different concentrations of Trolox (25–175 µM). The microplate was incubated at 30 °C for 5 min, and the absorbance of the resulting mixtures was measured at 734 nm.

##### ORAC Assay

The ORAC assay measured the antioxidant activity of the BSY fractions, following the protocol outlined by Dávalos et al. [43], with modifications. A stock solution of fluorescein disodium salt (MW = 376.27 g/mol) was prepared in phosphate buffer (PB) at a concentration of 75 mM and a pH of 7.4. A working solution of fluorescein was prepared at 116.66 mM. Phosphate buffer was used as the control and blank. A standard curve was generated using Trolox concentrations ranging from 10 to 80 µM. The assay was conducted in a 96-well microplate. The samples containing fluorescein were incubated and thoroughly mixed at 37 °C for 10 min. Subsequently, the AAPH solution (12 mM) was added rapidly to the standard, sample, and blank wells. The fluorescence was recorded at 1 min intervals over 90 min. The excitation wavelength was set to 485 nm, and the emission wavelength to 528 nm.

### 2.11. Crackers Preparation

The formulations of the crackers are presented in Table 1. The preparation followed the methodology described by Batista et al. [44], with modifications. The control formulation (C) consisted of 60.5% commercial all-purpose wheat flour T55 (composed of 76.7% carbohydrates, 7.8% protein, 2.9% fiber, and 1% lipids), 1.5% baking powder (corn starch, sodium diphosphate, and sodium bicarbonate), 1% salt (NaCl), 1% sugar (sucrose), 7.5% vegetable oil, and 28.5% distilled water.

Considering the objective of the VIIAFOOD project, in which this study is being developed, the formulation aimed to create prototypes enriched with alternative protein sources, and wheat germ (38.9%) was incorporated into the P0, P2, and P6 formulations as a high-protein ingredient, providing essential amino acids and functional properties beneficial for bakery applications [22]. Additionally, based on the well-documented functional and textural properties of tomato pomace in bakery products, 4% (*w*/*w*) of fine-ground tomato pomace flour (<250 µm) was added to the same formulations to enhance fiber content and improve water retention capacity [27].

The BSY fractions (P/RUF10) were incorporated into the P2 and P6 formulations at 2% and 6% (*w*/*w*), respectively, replacing the equivalent amounts of wheat flour. This fraction was selected due to its superior antioxidant capacity and peptide composition, as demonstrated in the results and discussion section below, making it the most suitable candidate for enhancing the functional properties of the crackers. The ingredients were weighed based on a 100 g batch and kneaded for 1 min at speed 4 in a food processor (Bimby, Vorwerk, Germany) to obtain a homogeneous, cohesive dough. For replications, three separate batches of each cracker formulation were prepared. The dough was further laminated into thin sheets, which were passed through a pasta roller machine under three different gauge positions (the dough was passed through one time for each position). A circle mold (6.8 cm) was used to cut the laminated dough into pieces, which were then slightly perforated. The shaped cracker dough was left to proof for 10 min at room temperature. The crackers were then baked in a forced-air convection oven (Unox, Italy) at 180 °C for 10 min. The baked samples were dried at 60 °C for 30 min, cooled at ambient temperature for 30 min, and stored in non-transparent closed plastic containers. Physical analyses were performed (color, texture, and water activity). Part of each cracker batch was immediately crushed to powder (using an electric mill) to be used for chemical analyses (biochemical composition and antioxidant capacity).

### 2.12. Chemical and Bioactive Properties of Crackers

The proximate composition, total dietary fibers (TDFs), macrominerals, total phenolic content (TPC), and antioxidant activity (AA) of the crackers were analyzed, following previously established methodologies. The energy content in each sample was calculated based on the Atwater factors using the following formula: (carbohydrates × 4.0 kcal/g) + (proteins × 4.0 kcal/g) + (lipids × 9.0 kcal/g) [3].

### 2.13. Physical and Technological Properties of Crackers

#### 2.13.1. Water Activity and Color Measurement

The water activity (a_w_) and color from the crackers prototypes were analyzed, following the previously established methodology [38].

#### 2.13.2. Density

The volume was determined, following the method of Mala et al. [38]. The density of the crackers was calculated and expressed as g/cm^3^.

#### 2.13.3. Texture

For texture analysis, a TA.XT.plus Texture Analyser (Stable MicroSystems) was used, with an HDP/BSG blade set with a guillotine. A 30 kg load cell was utilized, and the test was performed at 3 mm/sec, with a target distance of 10 mm and a trigger force of 50 g. The samples were placed on a platform with a slit and cut using the guillotine blade set, and the force was measured. The maximum exerted force was classified as hardness (N) and the distance at the break as fracturability (mm) [38].

#### 2.13.4. Scanning Electron Microscopy

The four fractured cracker samples were placed on top of the observation pins with the cross-section placed vertically (the cut was visible from above) and coated with gold/palladium. Their microstructure was analyzed using a Phenom XL G2 desktop scanning electron microscope (Thermo Fisher Scientific Inc., Waltham, MA, USA) at an accelerating voltage of 5 kV. Observations were performed, and micrographs were taken using a backscattered electron detector (BSD) [36].

### 2.14. Statistical Analysis

A one-way ANOVA was performed to detect significant differences between the six BSY extracts at both scales and the four crackers’ prototypes. Tukey’s test was posteriorly used for all pairwise means comparisons. The significance level was set at 5% in all the tests carried out. All the analyses were performed using Minitab^®^ 17.1.0 (LEAD Technologies, Inc., Charlotte, NC, USA). The data were expressed as the mean ± standard deviation.

## 3. Results and Discussion

### 3.1. Chemical Composition of the BSY By-Products

This study utilized three by-products: BSY, tomato pomace, and wheat germ. The results of the nutritional characterization of tomato pomace and wheat germ are presented in the Supplementary Materials, and this section focuses exclusively on the detailed characterization and processing of BSY. As the second most significant by-product of the brewing industry, BSY offers a rich composition that supports its application as a functional ingredient in food formulations [45]. Its chemical composition underscores its potential as a valuable ingredient in food and feed applications (Table 2).

Table 2 presents the values from the chemical composition analysis of the BSY by-products from beer production. Although the chemical composition of BSY is variable according to the brewing conditions and raw materials used, which differ among breweries, the results align with previous findings on BSY [7,46].

The results in Table 2 demonstrate that BSY is also a rich source of both macrominerals and trace elements, with high levels of potassium (6.29% of DRI per 100 g DW), phosphorus (30.36% of DRI per 100 g DW), and calcium (4.63% of DRI per 100 g DW), contributing to its nutritional value and supporting key functions, such as electrolyte balance, bone health, and energy metabolism. BSY also provides essential trace minerals, including iron (10.73% of DRI per 100 g DW for men), zinc (5.58% of DRI per 100 g DW for men), and copper (13.13% of DRI per 100 g DW for men), which play critical roles in enzymatic functions, immune responses, and antioxidant defense [5,47,48]. Consistent with the findings from Vieira et al. [49], the mineral content in BSY underscores its potential as a sustainable ingredient for developing fortified food products, addressing micronutrient deficiencies, and contributing to functional food formulations [50].

The comprehensive chemical characterization of BSY underscores its potential as a sustainable and functional ingredient for the food industry.

### 3.2. Evaluation of BSY Process Scale-Up Efficiency

The scale-up process plays a crucial role in industrial applications, as it enables the identification of key parameters that affect the retention of bioactive compounds. In this study, the scalability of BSY extraction and fractionation was evaluated by analyzing yield, protein content, peptide distribution, and antioxidant capacity at both laboratory and pilot scales. By comparing these parameters across different processing scales, this assessment provides valuable insights into how scale-up impacts the recovery, composition, and functional potential of bioactive peptides in food applications.

#### 3.2.1. BSY Peptide Profile

The molecular weight distribution of the BSY peptide fractions, analyzed by HPSEC, provides key insights into the composition and functional properties of the hydrolysates. The 10 kDa permeate fraction contained the highest proportion of small peptides (<1 kDa and 1–3 kDa), which are usually associated with their high solubility and bioactivity. The comparison between laboratory and pilot-scale fractions revealed that the retention of low-molecular-weight peptides was slightly reduced at the pilot scale, likely due to variations in transmembrane pressure, pore fouling, and shear stress during ultrafiltration [51]. These factors have been widely reported as critical parameters affecting the efficiency of membrane-based peptide separation [52].

**Figure 2:**
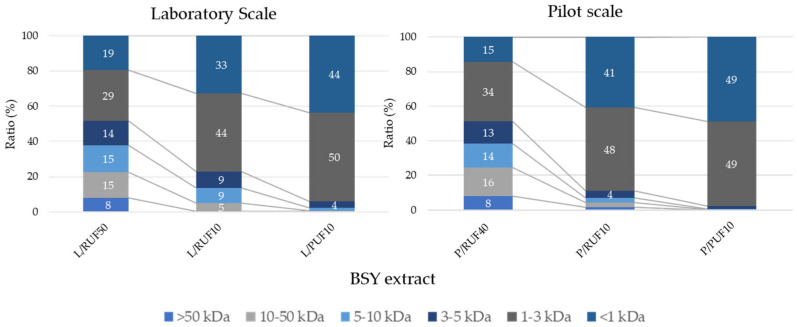
HPSEC of BSY extracts’ profiles on laboratory and pilot scales.

As shown in Figure 2, the laboratory-scale fractions exhibited a more defined molecular weight distribution, with a higher retention of peptides in the 1–3 kDa range. This suggests that small-scale ultrafiltration may allow for better control over processing conditions, preventing peptide aggregation and enhancing the retention of bioactive compounds. Amorim et al. [18] reported that membrane pore size and operational pressure significantly impact the fractionation of bioactive peptides, influencing their structural stability and functional properties.

In contrast, the pilot-scale permeate fractions displayed a broader distribution, indicating potential losses of low-molecular-weight peptides. This trend aligns with findings from recent studies on protein hydrolysate fractionation, where industrial-scale processes often experience challenges in maintaining consistent peptide profiles due to increased turbulence and non-uniform shear forces [53].

Further characterization of pilot-scale peptide fractions is necessary to optimize membrane performance and ensure consistency in bioactive peptide recovery during industrial-scale applications.

#### 3.2.2. Yield, Protein Content, Color Measurement, and Technological Properties

The yield percentages, protein content, color measurement, and technological properties (WHC and OHC) of the different BSY fractions obtained at both scales are presented in Table 3. Scaling up from 1 L (laboratory scale) to 100 L (pilot scale) resulted in variations in extraction yield, likely influenced by temperature control, stirring efficiency, and membrane performance. Firstly, the yield of each fraction was determined based on the total dry weight processed at each scale. The mass of the recovered fraction was divided by the initial dry weight of BSY processed at the laboratory (36.177 g DW) and pilot scales (29.956 kg DW) and expressed as a percentage (% DW). The fractionation yields differed significantly between the laboratory and pilot scales. At the laboratory scale, the highest yield was obtained in L/RUF50 (23.76%), followed by L/PUF10 (7.54%) and L/RUF10 (6.25%). These results suggest that the ultrafiltration process at a smaller scale retained a larger proportion of the high-molecular-weight compounds in the 50 kDa retentate (L/RUF50), while the lower-molecular-weight fractions were distributed among the L/RUF10 and L/PUF10 fractions. The yield differences between these fractions indicate an efficient separation of peptides and other soluble compounds across the different molecular weight cut-offs.

At the pilot scale, the highest yield was observed in P/RUF40 (17.77%), while P/RUF10 (0.70%) and P/PUF10 (0.08%) exhibited significantly lower yields. Compared to the laboratory-scale equivalent, P/RUF40 (40 kDa retentate) had a lower yield than L/RUF50 (50 kDa retentate), which may be due to differences in membrane selectivity and transmembrane pressure variations affecting retentate accumulation. Similarly, P/RUF10 and P/PUF10 showed much lower yields than their laboratory counterparts, indicating a reduced retention of low-molecular-weight compounds during large-scale ultrafiltration. A notable observation at the pilot scale was the clear loss of mass that was not observed at the laboratory scale. This loss of material could be attributed to handling procedures during pilot-scale processing, particularly during the initial autolysis phase, where mass loss may have occurred due to the use of only one decantation step and a manual transfer between the container and the membrane filtration system. These process differences likely contributed to the lower recovery of soluble fractions on the pilot scale.

When comparing equivalent fractions between scales, the retention of larger peptides in P/RUF40 was lower than in L/RUF50, suggesting that membrane performance, fouling, or operational conditions at a larger scale may have influenced the retention efficiency. Additionally, the P/RUF10 and P/PUF10 fractions exhibited drastic yield reductions compared to their laboratory-scale equivalents, potentially due to differences in shear forces, backpressure effects, and membrane fouling, leading to increased loss of low-molecular-weight peptides. Previous studies have demonstrated that optimizing transmembrane pressure and backwash sequences plays a crucial role in maintaining membrane efficiency and minimizing fouling during scale-up operations [54].

The protein content showed a significant difference between L/RUF50 and L/RUF10 across laboratory-scale fractions, with L/RUF10 exhibiting the highest protein concentration (31.12 ± 0.90%). However, at the pilot scale, the protein content was significantly lower, particularly in P/RUF10 (16.27 ± 0.51%), indicating potential protein losses or reduced retention efficiency in the ultrafiltration system. Similar results were observed in previous studies on protein hydrolysate fractionation, where scale-up often introduces variations in yield and protein retention due to changes in membrane dynamics, fluid shear forces, and operational stability, such as pH fluctuations and membrane material properties. Dullius et al. (2018) reported that scaling up whey protein hydrolysate fractionation resulted in a 15–20% decrease in protein retention due to increased shear stress and transmembrane pressure variations. Similarly, Vollet et al. (2021) demonstrated that pH strongly influences ultrafiltration performance in spent brewer’s yeast hydrolysates, with retention rates decreasing from 78% to 62% when the pH shifted from 6.5 to 4.0, highlighting the impact of pH-induced changes in protein solubility and membrane fouling. These findings reinforce the challenges associated with large-scale ultrafiltration and emphasize the need for optimized process conditions to maintain bioactive peptide retention and yield efficiency at an industrial scale [55,56]. Thus, as was mentioned above, the transition from laboratory- to pilot-scale processes often results in lower extraction efficiencies, attributed to losses in the tubular filtration system, differences in transmembrane pressure, pore fouling, and shear stress, which influence the selectivity and retention of bioactive compounds. Membrane performance at a larger scale is influenced by hydrodynamic conditions, backpressure, and fouling accumulation, which can hinder protein passage and reduce recovery rates [54]. Additionally, mass transfer limitations at larger volumes can reduce protein yield, particularly in ultrafiltration and diafiltration processes, as observed by Yadav et al. [51], where a 30% reduction in protein recovery was linked to increased turbulence and viscosity variations in pilot-scale hydrolysate processing. Furthermore, studies have shown that scale-up affects shear forces, which play a crucial role in protein conformation and bioactivity preservation. Excessive shear may lead to protein denaturation, potentially altering the functional and antioxidant properties of the hydrolysates. Similarly, the challenges of mass transfer efficiency and thermodynamic constraints during scale-up extraction have been widely discussed [57], emphasizing the need for parameter adjustments to ensure consistency in bioactive compound recovery.

The color parameters (L*, a*, and b*) indicate significant differences among the BSY fractions. The L* (lightness) values show that L/RUF10 (37.88 ± 2.54) and L/PUF10 (33.75 ± 2.14) exhibited the highest lightness, while P/RUF10 (20.99 ± 0.48) showed the lowest. This difference may be attributed to process-induced modifications, including oxidation levels and potential Maillard reactions [36] due to residual carbohydrate content, as previously observed in yeast-derived hydrolysates [18]. Furthermore, previous studies have shown that hydrolysates rich in low-molecular-weight peptides tend to exhibit darker coloration due to increased oxidative susceptibility and the formation of secondary oxidation products [58]. The observed trends suggest that processing conditions, including transmembrane pressure and flow rate, may play a role in maintaining the color stability of the BSY fractions.

The a* and b* values further support these differences, as L/PUF10 exhibited the highest redness (5.78 ± 0.14) and yellowness (12.69 ± 0.54), whereas P/RUF10 had the lowest values (2.53 ± 0.04 and 0.94 ± 0.16, respectively). This variation suggests that pilot-scale processing conditions, particularly pressure and filtration efficiency, may influence pigment retention and Maillard-derived coloration, as noted in hydrolysate studies [59]. Additionally, Amorim et al. [18] reported that yeast hydrolysates with a higher concentration of low-molecular-weight sugars exhibited increased browning intensity due to Maillard reactions.

The water-holding capacity (WHC) was only detected in P/RUF40 (1.12 ± 0.11 g water/g), while other fractions showed no measurable WHC values. This is likely due to a combination of factors, including high-molecular-weight peptides, a greater proportion of hydrophilic amino acids, and structural modifications during processing that enhance water retention. In contrast, smaller peptides in the other fractions were likely too short or too hydrophobic to effectively retain water. According to Amorim et al. [18], the amino acid profile of BSY hydrolyzed with enzymes varies based on molecular weight, with low-molecular-weight fractions containing higher levels of glutamic acid, glutamine, and alanine, which are known to enhance water retention. Additionally, glutamic acid and glutamine, which contribute to the umami flavor of hydrolysates, may also influence protein solubility and functional properties. This aligns with findings from Oliveira et al. [10], who reported that hydrophilic amino acids, such as arginine and aspartic acid, play a crucial role in protein hydration and gel-forming properties. Studies on fish and plant protein hydrolysates have also demonstrated that smaller peptides and amino acids contribute to lower WHC values, while larger, more structured proteins retain better water-binding ability [58].

The oil-holding capacity (OHC) varied significantly among the BSY fractions, with L/RUF50 exhibiting the highest OHC (4.08 ± 0.23 g oil/g), followed by L/RUF10 (3.36 ± 0.62 g oil/g) and L/PUF10 (3.15 ± 0.75 g oil/g), whereas P/RUF10 (0.45 ± 0.14 g oil/g) displayed the lowest capacity. These differences can be attributed to the presence of hydrophobic amino acids and the structural characteristics of peptides, which influence their interaction with lipid molecules, a key factor affecting oil retention in protein-based food systems. Oliveira et al. reported that hydrolysates with higher-molecular-weight fractions exhibit increased OHC due to their structural conformation, which enhances their lipid-binding capacity [10]. In contrast, the substantial reduction in OHC observed in pilot-scale fractions (P/RUF40, P/RUF10, and P/PUF10) may result from membrane-induced structural modifications, leading to alterations in hydrophobic surface exposure or the loss of lipid-binding peptides during processing. Furthermore, Amorim et al. (2016) highlighted that enzymatic hydrolysis conditions and membrane filtration significantly affect the emulsification properties of protein-rich fractions, subsequently impacting their oil absorption ability [18]. These findings suggest that optimizing hydrolysis and filtration conditions at the pilot scale could improve the functional properties of BSY-derived fractions, particularly their emulsifying and lipid-binding potential, making them valuable for food applications where fat retention and texture stability are critical.

#### 3.2.3. Total Phenolic Content and Antioxidant Capacity

**Figure 3:**
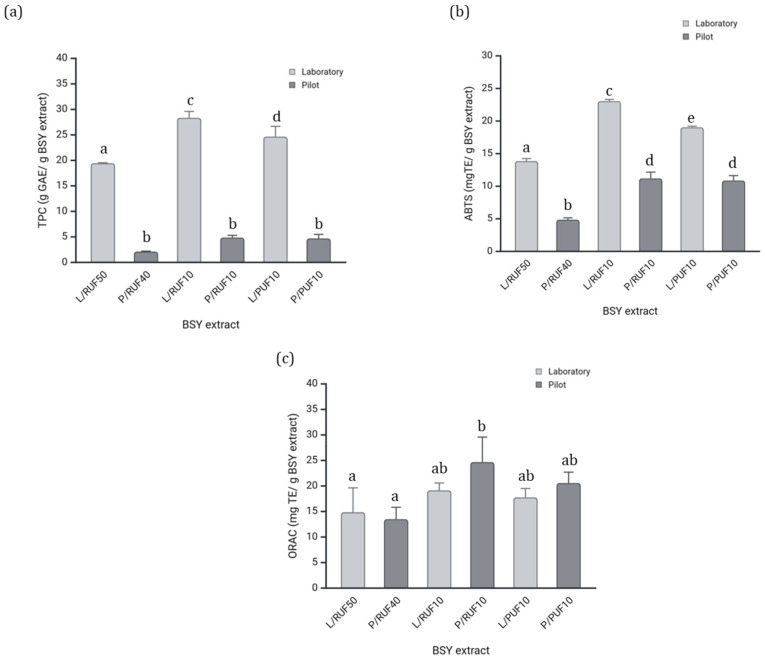
(**a**) TPC and AA, (**b**) ABTS, and (**c**) ORAC assays of the different BSY extracts in both scales (laboratory and pilot). The data is shown as the mean ± SD from three replicates. The different letters represent the significant difference at *p* < 0.05.

The assessment of TPC and AA in BSY extracts provides critical insights into the retention and stability of bioactive compounds during scale-up processing. These parameters were evaluated using the TPC, ABTS, and ORAC assays to compare the functional properties of fractions obtained at laboratory and pilot scales. The results exhibited significant differences between both scales, particularly in the ABTS and TPC assays, as shown in Figure 3.

The TPC results (Figure 3a) demonstrated that L/RUF10 and L/PUF10 showed significantly higher phenolic content compared to the pilot-scale fractions. The discrepancy in phenolic retention may be due to variations in membrane efficiency and process scale, as reported by Hamzah and Leo [60], which demonstrated that pressure-driven membrane filtration processes significantly impact phenolic compound stability due to variations in transmembrane pressure and fouling mechanisms. Similarly, Conidi et al. [61] found that nanofiltration membranes lead to higher phenolic retention compared to ultrafiltration, reinforcing the importance of process parameter optimization for maximizing bioactive compound recovery.

Regarding the ABTS assay (Figure 3b), the results also revealed significant differences (*p* < 0.05) between the laboratory and pilot scales. L/RUF10 exhibited the highest ABTS scavenging activity, significantly surpassing its pilot-scale counterpart (P/RUF10). These results are aligned with the findings observed in the TPC (Figure 3a). The strong correlation between the TPC and ABTS antioxidant activity is closely linked to the structural characteristics of phenolic compounds. Another study [62] demonstrated that phenolic acids interact with ABTS radicals, primarily through hydrogen bonding and electron transfer mechanisms, with hydroxyl and carboxyl groups playing a key role in antioxidant activity. Similarly, Choi et al. [63] found that increased phenolic content significantly enhances ABTS radical neutralization, further supporting the role of phenolic compounds as key contributors to antioxidant mechanisms.

The ORAC assay (Figure 3c) indicated that P/RUF10 (pilot-scale retentate, 10 kDa) exhibited the highest antioxidant activity, followed closely by L/RUF10 (laboratory-scale retentate, 10 kDa). The increase in pilot samples roasted could be caused by the contribution of other compounds (like proteins, peptides, or aromatic components). This suggests that low-molecular-weight peptides and phenolic compounds contribute significantly to the antioxidant potential. These findings are consistent with those of Amorim et al. [18], who demonstrated that low-molecular-weight peptide-rich ultrafiltration fractions maintain high antioxidant activity across different processing conditions.

Based on the comprehensive characterization of the fractions, we conclude that the properties were generally maintained between laboratory and pilot scales, with some variations attributed to process scale-up effects. While both P/RUF40 and P/RUF10 exhibited viable characteristics for cracker formulation, P/RUF10 was ultimately selected due to a combination of factors. Although the total phenolic content and antioxidant activity were comparable between P/RUF10 and P/PUF10, P/RUF10 demonstrated a significantly higher ORAC value, indicating greater radical scavenging potential. Additionally, P/RUF10 exhibited a substantially higher yield than P/PUF10 (Table 3), making it a more practical choice for application. This selection ensures that the final product benefits from enhanced functional properties while maintaining feasibility for large-scale implementation.

### 3.3. Properties of Crackers with BSY Fraction

This discussion will primarily focus on the impact of BSY incorporation on the physicochemical and bioactive properties of the crackers. However, it is important to note that the characterization results presented in Table 4 already indicate a significantly high level of ash, protein, and total dietary fiber (TDF) content in the P0 formulation, even before BSY addition [22,27]. This reflects the partial substitution of wheat flour with tomato pomace and wheat germ, both of which are known for their high fiber and protein content. The presence of these ingredients in P0 establishes a baseline nutritional enhancement, against which the effects of BSY incorporation can be further evaluated.

#### 3.3.1. Chemical Analysis of Crackers

The proximate composition and bioactive properties of the BSY-enriched crackers are summarized in Table 4. The incorporation of BSY, tomato pomace, and wheat germ significantly altered the nutritional profile, leading to increased protein and fiber content while modifying the energy and fat content.

The moisture content ranged from 13.49 ± 1.97% (P0) to 19.79 ± 6.97 (P6), with no significant differences among the samples. The slightly higher moisture content in P6 could be due to the increased protein and fiber content, which can enhance water retention, as observed in high-protein bakery formulations [27,44]. The ash content significantly increased with BSY inclusion, reaching the peak in P2 (3.41 ± 0.06), reflecting the contribution of minerals from yeast and wheat germ, as reported in previous studies [38].

The protein content significantly increased in all the BSY-enriched crackers compared to the control (C), with P6 exhibiting the highest protein concentration (26.52 ± 1.77%), which aligns with our characterization and previous findings indicating that BSY is a rich protein source [10]. Similar increases in protein content have been reported in alternative protein-enriched crackers [13]. The incorporation of BSY fractions also influenced the fat content, with P6 showing the lowest fat concentration (3.05 ± 0.10%), potentially due to protein interactions limiting fat retention [18]. Despite these compositional shifts, the energy values remained statistically similar across all formulations (*p* > 0.05), suggesting that BSY integration does not compromise the caloric density of the product.

Regarding carbohydrate content, a significant decrease was observed in the BSY-enriched samples, likely due to protein substitution in the formulation. P6 had the lowest carbohydrate content (58.88 ± 0.28%), further confirming the role of protein-rich fractions in reducing overall carbohydrate levels. This aligns with findings from Mala et al. [38], who reported similar reductions in carbohydrate content in high-protein functional crackers.

The TDF content significantly increased in the BSY-enriched samples, reaching 30.51 ± 0.02 g/100 g in P6. Yeast-derived β-glucans have been shown to contribute to fiber content, but their impact alone would not account for the observed increase [14]. The higher fiber content observed in P6 is beneficial, as it contributes to improved gastrointestinal health and potential cholesterol-lowering effects, aligning with previous studies on high-fiber functional foods [64,65].

Additionally, mineral analysis revealed a notable increase in phosphorus (P), magnesium (Mg), and potassium (K) levels in the BSY-enriched formulations, in accordance with the initial BSY characterization. The higher phosphorus content in P6 (658 ± 1.80 mg/100 g DW) aligns with the phosphorus-rich profile of BSY, contributing to essential metabolic functions and bone health. The higher phosphorus content in P6 (658 ± 1.80 mg/100 g DW) aligns with the phosphorus-rich profile of BSY, contributing to essential metabolic functions and bone health [66]. Considering the daily recommended intake (DRI) of P is 550 mg/day (according to EFSA), a 100 g portion of P6 provides over 100% of the daily requirement, highlighting its relevance as a mineral-rich ingredient. Similarly, Mg levels in P6 (125 ± 0.20 mg/100 g DW) represent 35–41% of the DRI, reinforcing its potential role in muscle contraction, nerve signaling, and enzymatic reactions. K levels (588 ± 0.84 mg/100 g DW) in P6 contribute approximately 17% of the daily requirement, supporting cardiovascular function and electrolyte balance, further supporting the potential benefits of BSY as a functional ingredient in food formulations [67]. On the other hand, sodium (Na) levels remained relatively unchanged across formulations (≈750 mg/100 g DW), suggesting that the incorporation of BSY, wheat germ, and tomato pomace did not significantly influence the overall Na content. This is particularly relevant for product formulation, as maintaining Na levels within moderate ranges is essential for meeting consumer health preferences, especially given the association between excessive Na intake and cardiovascular risk [68].

A significant increase in total phenolic content (TPC) was observed in the BSY-enriched crackers, with P6 showing the highest phenolic concentration (329.3 ± 18.7 mg GAE/100 g DW). Notably, crackers containing tomato pomace and wheat germ (P0) already exhibited an increase in TPC compared to the control sample, suggesting that these ingredients contribute significantly to the phenolic content. However, the inclusion of BSY in P2 and P6 further enhanced TPC levels, although the differences were not statistically significant. This trend indicates that BSY contributes to enrichment in bioactive compounds, likely through the presence of bioactive peptides and yeast-derived phenolics, which have been reported in previous studies [69]. This increase correlates strongly with the enhanced antioxidant capacity measured by the ABTS assays, where P6 also exhibited the highest radical scavenging activity (20.10 ± 0.55 g TE/100 g DW). The ABTS results followed a similar trend to TPC, though a significantly greater difference (*p* < 0.05) was observed between P0 and the BSY-enriched crackers (P2 and P6). This suggests that antioxidant activity results from the combined contribution of phenolic compounds and bioactive peptides present in the BSY fraction, reinforcing the functional benefits of selecting P/RUF10 for the cracker formulation. These results agree with [49], which demonstrated that BSY-derived peptides and phenolics significantly contribute to antioxidant activity. The observed increase in TPC suggests that BSY inclusion in crackers enhances their functional potential.

Overall, BSY incorporation led to significant improvements in protein, fiber, and antioxidant content, while maintaining energy levels comparable to traditional crackers. The increase in phenolic compounds and ABTS activity suggests that BSY-enriched crackers could serve as functional snacks with enhanced nutritional value.

#### 3.3.2. Physical Characteristics of Crackers

The physical and structural characteristics of the crackers were influenced by the inclusion of BSY, as shown in Table 4. The water activity (a_w_) values significantly decreased with the addition of BSY, particularly in the P6 sample (a_w_ = 0.60 ± 0.04). The findings revealed significant differences between the crackers with BSY inclusion and the control ones, which could be attributed to the higher protein content and fiber incorporation, both of which can interact with water molecules and reduce free water availability, as previously observed in fiber-enriched crackers [38]. This reduction in water activity can positively impact shelf stability by limiting microbial growth, a key factor in baked products.

**Figure 4:**
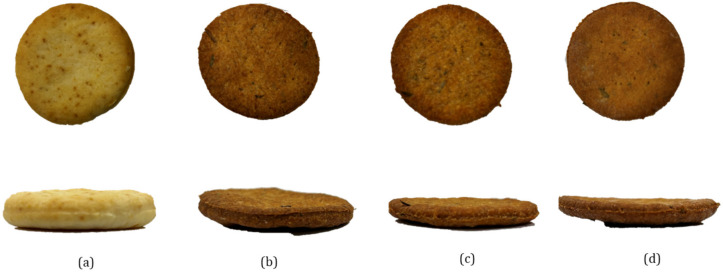
Appearance of the crackers from the top and front view: (**a**) control sample (C), (**b**) sample with 0% BSY (P0), (**c**) sample with 2% BSY (P2), and (**d**) sample with 6% BSY (P6).

The color parameters revealed significant changes with BSY, tomato pomace, and wheat germ incorporation, as presented in Figure 4. The lightness (L*) values decreased drastically in the new-by-products-enriched samples, particularly in P0, P2, and P6, aligning with findings from Batista et al. and Nakov et al. [27,44], who reported a similar effect when incorporating microalgae and tomato pomace into cracker formulations, respectively. The increased redness (a*) and yellowness (b*) in the BSY-enriched samples are consistent with the presence of Maillard reaction products and caramelization due to protein and fiber interactions at high baking temperatures. The lower L* values and higher a* values may be related to the development of melanoidins, known for their antioxidant properties, as documented in previous studies on functional cracker formulations [27,38]. The increase in the a* value may also be attributed to the presence of lycopene in tomato pomace, a carotenoid responsible for the red coloration, which has been shown to enhance the red hue in bakery products enriched with tomato-derived ingredients [70].

Dimensional properties, including diameter and thickness, did not show significant differences among the samples, suggesting that BSY inclusion did not negatively affect the spreadability of the dough. However, the weight and density exhibited a decreasing trend in the P6 sample, which could be related to the lower moisture retention upon baking, an effect previously reported in protein- and fiber-enriched crackers [44]. This reduction in weight and density is desirable from a consumer standpoint, as lighter crackers are often associated with a crispier texture.

Texture analysis revealed significant differences in hardness and fracturability among samples. The control sample (C) exhibited the lowest hardness (68.40 ± 10.94 N), while the BSY-enriched samples, particularly P0 and P2, demonstrated increased hardness. This agrees with previous studies on fiber-enriched bakery products, where fiber–protein interactions contributed to a firmer texture [38]. Interestingly, P6 showed a drastic decrease in hardness, which could be attributed to disruptions in the gluten matrix due to excessive protein and fiber incorporation, leading to a more brittle structure. The fracturability values further support this observation, as P2 exhibited the highest fracturability (6.57 ± 1.06 mm), suggesting improved crispness, a key sensory attribute in cracker formulations.

The visual appearance of the crackers, depicted in Figure 4, highlights the progressive darkening effect with increasing BSY content, consistent with the colorimetric analysis. The flatter structure of the P6 sample corresponds with their lower density and higher fracturability, reinforcing the impact of BSY on textural properties. The changes in appearance and structure are similar to those reported in functional cracker formulations enriched with alternative protein sources [38,44], further supporting the feasibility of BSY as a functional ingredient.

**Figure 5:**
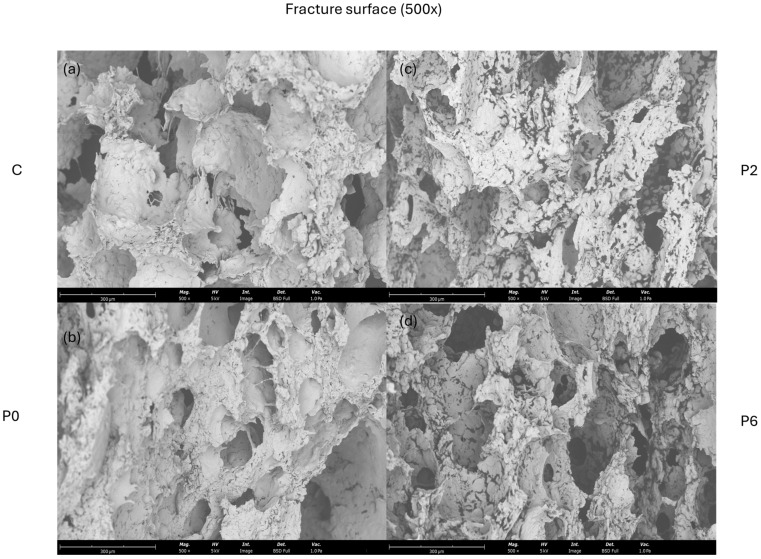
SEM micrographs of the crackers from the fracture surface view (500×): (**a**) control sample (C), (**b**) sample with 0% BSY (P0), (**c**) sample with 2% BSY (P2), and (**d**) sample with 6% BSY (P6).

The SEM micrographs in Figure 5 illustrate the fracture surface morphology of the four different cracker samples, highlighting the structural differences influenced by the inclusion of BSY, wheat germ, and tomato pomace. The control sample (C) exhibited a relatively compact and uniform structure, indicative of a well-developed gluten-starch network. In contrast, the P0 sample, which includes wheat germ and tomato pomace without BSY, showed a more disrupted network with visible fractures, consistent with the findings of Mala et al. [38], where the addition of dried pineapple peel powder resulted in surface cracks due to the disruption of the gluten–starch matrix.

As BSY levels increased in the P2 and P6 samples, the micrographs revealed a more open and porous structure. This observation aligns with the introduction of fiber-rich and protein-based ingredients, which are known to interfere with the formation of a cohesive gluten network. This effect has been documented in other studies, such as Dachana et al. [71], who reported that higher levels of plant-based ingredients, like dried Moringa leaf powder, led to a greater quantity of pores and cracks in the product’s structure.

The porous microstructure observed in the P6 sample is likely due to the higher BSY content, which introduced peptides and polysaccharides, weakening the overall structural cohesiveness of the dough.

BSY incorporation effectively enhanced the protein, fiber, and antioxidant properties of the crackers while modifying their physical attributes. Although these modifications were largely beneficial, further optimization is required to balance functional improvements. These findings underscore BSY’s potential as a sustainable and nutritionally enriching ingredient in bakery applications, aligning with the increasing demand for functional food products.

## 4. Conclusions

The comprehensive chemical characterization of BSY underscores its potential as a sustainable and functional ingredient for the food industry. Its high protein and carbohydrate content, alongside peptides with antioxidant activity and essential minerals, makes it a promising candidate for developing functional foods and nutraceuticals.

The successful transition to pilot-scale production supports the feasibility of integrating BSY-derived fractions into food formulations, particularly protein-enriched ones, here demonstrated with crackers, where 2% and 6% BSY fractions were tested. However, pilot-scale processes retained antioxidant activity but showed lower protein and phenolic compound recovery compared to laboratory-scale fractions. Process conditions must be optimized to enhance peptide retention and antioxidant stability in larger-scale applications.

This study also demonstrated that the incorporation of BSY fractions into cracker formulations effectively enhanced their nutritional composition and bioactive properties. The inclusion of BSY significantly increased the protein and fiber content in the crackers, particularly in the P6 formulation, aligning with the functional potential of yeast-derived ingredients. Additionally, the mineral content, particularly phosphorus, magnesium, and potassium, was positively influenced by BSY addition, reinforcing its role as a valuable source of essential macronutrients.

The antioxidant potential of BSY-enriched crackers was confirmed through the TPC and ABTS assays, where a progressive increase in phenolic content and radical scavenging activity was observed. Additionally, the ORAC assay results demonstrated that P/RUF10 exhibited the highest antioxidant activity, closely followed by L/RUF10. This tendency indicates that low-molecular-weight peptides and phenolic compounds are key contributors to the antioxidant potential of BSY fractions. The observed trends validate the selection of the P/RUF10 fraction, which exhibited superior antioxidant activity, making it the most suitable fraction for functional bakery applications.

Physicochemical characteristics such as color, water activity, and texture were influenced by the BSY addition. The decrease in L* values and increase in a* values indicate the formation of Maillard reaction products, likely enhanced by the presence of residual sugars in BSY, as previously reported in the literature. The reduction in water activity in the BSY-enriched samples suggests potential improvements in shelf stability, while the observed differences in textural parameters highlight the importance of optimizing BSY inclusion to maintain desirable structural properties.

Overall, BSY shows great promise as a sustainable and functional ingredient in bakery applications, contributing to circular economy principles by valorizing the brewing industry’s by-products. Future work should focus on exploring its sensorial properties and bioavailability to maximize its potential applications.

## Figures and Tables

**Table 1:** The dough formula of the crackers with different levels of BSY fraction.

Ingredients	C (%)	P0 (%)	P2 (%)	P6 (%)
Wheat Flour	60.5	24.0	22.0	18.0
Baking Powder	1.5	1.5	1.5	1.5
Salt	1.0	1.0	1.0	1.0
Sugar	1.0	1.0	1.0	1.0
Vegetable Oil (sunflower oil)	7.5	7.5	7.5	7.5
Water	28.5	22.0	22.0	22.0
Tomate Pomace Flour (<250 µm)		4.0	4.0	4.0
Thyme		0.1	0.1	0.1
P/RUF 10			2.0	6.0
Wheat Germ		38.9	38.9	38.9

**Table 2:** Chemical composition of the initial BSY by-products.

Chemical Composition (g/100 g DW)		BSY
Proximate composition	Moisture	84.1 ± 0.05
	Ash	5.52 ± 0.02
	Proteins	43.6 ± 0.45
	Fat	2.51 ± 0.01
	Carbohydrates	48.4 ± 0.48
	Energy ^a^	390.59
Macrominerals (mg/100 g DW)	Ca	43.9 ± 6.48
	K	220 ± 2.70
	Na	2.92 ± 0.06
	Mg	27.3 ± 1.69
	P	167 ± 5.71
Trace minerals (mg/100 g DW)	Al	0.02 ± 0.01
	Cu	0.21 ± 0.00
	Fe	1.18 ± 0.09
	Mn	0.06 ± 0.01
	Zn	0.53 ± 0.04

Results are expressed as mean values ± SD from three replicates. ^a^ kcal/100 g.

**Table 3:** The yield percentages, protein content, color measurement parameters, and technological properties values of the different BSY fractions.

Parameters	L/RUF50	L/RUF10	L/PUF10	P/RUF40	P/RUF10	P/PUF10
Yield (% DW)	23.76 ^a^	6.25 ^b^	7.54 ^c^	17.77 ^d^	0.70 ^e^	0.08 ^f^
Protein (% DW)	27.06 ± 0.92 ^a^	31.12 ± 0.90 ^b^	28.5 ± 0.39 ^ab^	22.55 ± 2.85 ^c^	16.27 ± 0.51 ^d^	18.01 ± 1.18 ^d^
L*	24.29 ± 0.63 ^bc^	37.88 ± 2.54 ^a^	33.75 ± 2.14 ^a^	24.16 ± 0.99 ^bc^	20.99 ± 0.48 ^c^	25.87 ± 2.33 ^b^
a*	4.14 ± 0.20 ^bc^	4.92 ± 0.33 ^b^	5.78 ± 0.14 ^a^	3.93 ± 0.13 ^c^	2.53 ± 0.04 ^d^	3.60 ± 0.61 ^c^
b*	7.16 ± 0.31 ^c^	10.79 ± 0.66 ^b^	12.69 ± 0.54 ^a^	6.90 ± 0.21 ^c^	0.94 ± 0.16 ^e^	2.56 ± 0.44 ^d^
WHC (g water/g)	ND ^a^	ND ^a^	ND ^a^	1.12 ± 0.11 ^b^	ND ^a^	ND ^a^
OHC (g oil/g)	4.08 ± 0.23 ^a^	3.36 ± 0.62 ^ab^	3.15 ± 0.75 ^b^	1.35 ± 0.02 ^c^	0.45 ± 0.14 ^c^	1.02 ± 0.14 ^c^

Results are expressed as the mean values ± SD from three replicates. The different letters in the same row represent the significant difference at *p* < 0.05. ND, not detected.

**Table 4:** Proximate composition (g/100 g DW), chemical, bioactive, and physical characteristics of the prepared crackers.

Parameters	C (%)	P0 (%)	P2 (%)	P6 (%)
Moisture (%)	15.77 ± 6.95 ^a^	13.49 ± 1.97 ^a^	18.07 ± 0.95 ^a^	19.79 ± 6.97 ^a^
Ash (%)	1.94 ± 0.01 ^c^	3.30 ± 0.04 ^b^	3.41 ± 0.06 ^a^	3.05 ± 0.10 ^b^
Protein (%)	10.39 ± 0.61 ^b^	23.61 ± 1.05 ^a^	24.88 ± 1.31 ^a^	26.52 ± 1.77 ^a^
Fat (%)	9.52 ± 0.14 ^d^	14.01 ± 0.14 ^a^	9.71 ± 0.07 ^c^	3.05 ± 0.10 ^b^
Carbohydrates (%)	78.15 ± 0.75 ^a^	59.07 ± 0.59 ^c^	62.00 ± 0.52 ^b^	58.88 ± 0.28 ^c^
Energy (kcal/100 g DW)	439.84 ± 6.69 ^a^	456.85 ± 7.83 ^a^	434.91 ± 7.96 ^a^	445.56 ± 10.76 ^a^
TDF (g/100 g DW)	13.85 ± 0.55 ^c^	26.19 ± 0.05 ^b^	26.99 ± 0.09 ^b^	30.51 ± 0.02 ^a^
P (mg/100 g DW)	237.52 ± 2.04 ^b^	636.00 ± 0.74 ^a^	655.00 ± 1.17 ^a^	658.00 ± 1.80 ^a^
P (%DRI/100 g DW)	33.93 ± 0.04 ^b^	90.86 ± 0.14 ^a^	93.57 ± 0.17 ^a^	94.00 ± 0.80 ^a^
Mg (mg/100 g DW)	ND ^c^	122.00 ± 0.14 ^b^	116.00 ± 0.31 ^b^	125.00 ± 0.20 ^a^
Mg (%DRI/100 g DW)	ND ^c^	34.86 ± 0.04 ^b^	33.14 ± 0.01 ^b^	35.71 ± 0.02 ^a^
Na (mg/100 g DW)	735.00 ± 1.15 ^a^	754.00 ± 0.92 ^a^	746.00 ± 1.42 ^a^	754.00 ± 2.39 ^a^
Na (%DRI/100 g DW)	49.00 ± 0.15 ^a^	50.27 ± 0.22 ^a^	49.73 ± 0.42 ^a^	50.27 ± 0.39 ^a^
K (mg/100 g DW)	171.83 ± 1.58 ^c^	577.00 ± 0.22 ^b^	570.00 ± 0.17 ^b^	588.00 ± 0.84 ^a^
K (%DRI/100 g DW)	4.91 ± 0.08 ^c^	577.00 ± 0.22 ^b^	570.00 ± 0.17 ^b^	588.00 ± 0.84 ^a^
TPC (mg GAE/100 gDW)	54.70 ± 19.40 ^b^	16.49 ± 2.10 ^a^	16.29 ± 1.40 ^a^	16.80 ± 1.70 ^a^
ABTS (g TE/100 gDW)	4.93 ± 0.96 ^c^	13.35 ± 2.63 ^b^	18.59 ± 1.32 ^a^	20.10 ± 0.55 ^a^
Water Activity (a_w_)	0.76 ± 0.07 ^a^	0.64 ± 0.05 ^ab^	0.68 ± 0.03 ^ab^	0.60 ± 0.04 ^b^
L*	86.97 ± 4.05 ^a^	54.81 ± 1.89 ^b^	61.08 ± 1.34 ^b^	56.45 ± 2.35 ^b^
a*	5.39 ± 0.33 ^a^	11.52 ± 1.97 ^b^	11.38 ± 0.86 ^b^	11.01 ± 1.28 ^b^
b*	20.73 ± 0.25 ^bc^	23.15 ± 0.91 ^ab^	24.57 ± 1.50 ^a^	19.60 ± 0.86 ^c^
Diameter (cm)	5.57 ± 0.27 ^a^	5.47 ± 0.15 ^a^	5.99 ± 0.21 ^a^	5.80 ± 0.22 ^a^
Thickness (cm)	0.85 ± 0.09 ^a^	0.61 ± 0.13 ^ab^	0.75 ± 0.12 ^ab^	0.55 ± 0.06 ^b^
Weight (g)	10.63 ± 0.60 ^a^	8.753 ± 0.87 ^ab^	10.12 ± 2.39 ^ab^	7.12 ± 0.33 ^b^
Density (g/cm^3^)	0.44 ± 0.05 ^a^	0.37 ± 0.06 ^ab^	0.36 ± 0.07 ^ab^	0.27 ± 0.01 ^b^
Hardness (N)	68.40 ± 10.94 ^a^	108.20 ± 27.20 ^a^	94.60 ± 28.60 ^a^	115.24 ± 11.70 ^a^
Fracturability (mm)	2.18 ± 0.50 ^b^	2.25 ± 1.17 ^b^	6.57 ± 1.06 ^a^	3.15 ± 0.23 ^b^

Results are expressed as the mean values ± SD from three replicates. The different letters in the same row represent the significant difference at *p* < 0.05. ND, not detected.

## Data Availability

The original contributions presented in this study are included in the article/Supplementary Materials. Further inquiries can be directed to the corresponding author.

## Supplementary Materials

The following supporting information can be downloaded at: https://www.mdpi.com/article/10.3390/foods14071144/s1. Table S1. Tomato Pomace by-product Granulometry Analysis; Table S2. Chemical composition of the Tomato Pomace (TB) < 250 µm fraction and Wheat Germen (WG) by-products.

## Abbreviations

The following abbreviations are used in this manuscript:

AA	Antioxidant activity
AAPH	2,20-azobis (2-methylpropionamidine) dihydrochloride
ABTS	Diammonium salt (2, 2-azinobis-3-ethylbenzothiazoline-6-sulfonic acid)
AOXs	Antioxidant assays
BSY	Brewer’s spent yeast
DW	Dry weight
GAEs	Gallic acid equivalents
HPSEC	High-Performance Size Exclusion Chromatography
KDa	Kilodaltons
L/PUF10	Permeate from 10 kDa ultrafiltration at laboratory scale
L/RUF10	Permeate from 50 kDa/retentate from 10 kDa ultrafiltration at laboratory scale
L/RUF50	Retentate from 50 kDa ultrafiltration at laboratory scale
MWCO	Molecular weight cut-off
OHC	Oil-holding capacity
ORAC	Oxygen radical absorbance capacity
P/PUF10	Permeate from 10 kDa ultrafiltration at pilot scale
P/RUF10	Permeate from 40 kDa/retentate from 10 kDa ultrafiltration at pilot scale
P/RUF40	Retentate from 40 kDa ultrafiltration at pilot scale
PB	Phosphate buffer
PTFE	Polytetrafluoroethylene
TDF	Total dietary fiber
TEs	Trolox equivalents
TPC	Total phenolic content
UF	Ultrafiltration
WHC	Water-holding capacity
