# Supplementary material for: Peptide-Rich Yeast Fractions from Brewer’s Spent Yeast: A Scalable Fractionation Approach and Their Functional Application in Bakery Products
Source: Foods. 2025 Mar 25;14(7):1144. doi: 10.3390/foods14071144 (PMC11988475; doi:10.3390/foods14071144)
Supplement: Supplementary file 1 [file foods-14-01144-s001.zip › foods-3536669-supplementary.pdf]

Article

Peptide-Rich Yeast Fractions from Brewer's Spent Yeast: A Scalable Fractionation Approach and Their Functional Application in Bakery Products

María Emilia Brassesco [1,*,†], Ana Paupério [1,†], Carlos D. Pereira [2], João Paulo Ferreira [1] and Manuela Pintado [1]

[1] CBQF—Centro de Biotecnologia e Química Fina, Laboratório Associado, Escola Superior de Biotecnologia, Universidade Católica Portuguesa, Rua Diogo Botelho 1327, 4169-005 Porto, Portugal; airibeiro@ucp.pt (A.P.); jpferreira@ucp.pt (J.P.F.); mpintado@ucp.pt (M.P.)
[2] Politécnico de Coimbra, Escola Superior Agrária, Bencanta, 3045-601 Coimbra, Portugal; cpereira@esac.pt
* Correspondence: mbrassesco@ucp.pt
† Joint first authorship is being claimed. These authors contributed equally to this work.

Supplementary Materials

1.1. Granulometry Analysis of Tomato Pomace by-product.

The particle-size distributions of Tomato Pomace (TB) were assessed by using an automated sieve shaker (Retsch AS 200, Haan, Germany) featuring circular oscillation. This analysis involved the use of 50 g of flour, a sifting duration of 5 min, and an amplitude of 1.85. Four sieves, each with a diameter of 20 cm and mesh sizes of 50, 100, 150, 250, 500, and 1000 μm, were employed for this purpose.

Table S1. Tomato Pomace by-product Granulometry Analysis.

Fraction	% relative
**50-100 μm**	0.26 ± 0.07
**100-150 μm**	5.93 ± 1.47
**150-250 μm**	19.86 ± 1.51
**250-500 μm**	**42.49 ± 1.30 ***
**500 μm-1 mm**	31.33 ± 1.41
**> 1 mm**	0.12 ± 0.01
*** predominant fraction**	

1.2. Nutritional Composition of Tomato Pomace and Wheat Germen by-product.

Table S2. Chemical composition of the Tomato Pomace (TB) <250 μm fraction and Wheat Germen (WG) by-products.

**Chemical Composition (g/100g DW)**		**TP**	**WG**
proximate Composition	Moisture	4.4 ± 0.1	12.8 ± 0.3
	Ash	3.9 ± 0.1	4.3 ± 0.0
	Proteins	20.5 ± 0.2	33.8 ± 0.7
	Lipids	11.3 ± 0.0	9.7 ± 0.0
	Carbohydrates	64.3 ± 0.0	52.2 ± 0.7
	Total Dietary Fiber	71.8 ± 1.05	11.2 ± 0.2
	Energy [a]	440.9	432.5

Results are expressed as mean values ± SD from three replicates. [a] kcal/100 g.
